# Influence of Drying Technique on Physicochemical Properties of Synthetic Hydroxyapatite and Its Potential Use as a Drug Carrier

**DOI:** 10.3390/ma16196431

**Published:** 2023-09-27

**Authors:** Karina Niziołek, Dagmara Słota, Julia Sadlik, Emilia Łachut, Wioletta Florkiewicz, Agnieszka Sobczak-Kupiec

**Affiliations:** Department of Materials Science, Faculty of Materials Engineering and Physics, Cracow University of Technology, 37 Jana Pawła II Av., 31-864 Krakow, Poland

**Keywords:** ceramic, hydroxyapatite, drug delivery system, clindamycin

## Abstract

Naturally occurring hydroxyapatite (HA) is the mineral phase of bone tissue. It is characterized by its bioactivity toward stimulating bone cells to proliferate and thus form new apatite layers. For this reason, it is a material commonly used in implantology for filling defects or covering implants (such as endoprostheses). There are several methods to obtain synthetic HA, and by controlling parameters such as temperature, pressure or the drying process, physicochemical parameters of the final powder can be affected. In the present study, HA was obtained by wet precipitation technique and subjected to two different drying methods, determining whether this parameter significantly affects the properties of the final material obtained. Analyzed Fourier-transform infrared spectroscopy (FT-IR) confirmed the presence of functional groups typical for HA. X-ray diffraction analysis (XRD) demonstrated that the materials are partially amorphous; however, the only phase was identified in HA. Scanning electron microscopy (SEM) was used to evaluate the surface morphology and the density, and average grain diameter was measured. Furthermore, HA powders were subjected to modification with the antibiotic clindamycin to determine the potential for use as a carrier for the active substance. The release rate of the drug was determined by high-performance liquid chromatography (HPLC). The differences in the characteristics of the powders were relatively small; however, they affected the rate of drug release from the material as well as the shape of the grains. The method of drying the powders was shown to affect the shape of the grains, as well as the porosity of the sinters prepared from it. A higher amount of clindamycin released into PBS was observed in material with more pores. The materials have demonstrated the potential to be used as a carrier for the active substance; however, further biological, as well as physicochemical, analysis is required.

## 1. Introduction

One of the modern solutions for treating diseases is controlled drug delivery systems (DDS). When drugs are administered conventionally over a long period of time, this can result in uneven distribution in the body. These allow the delivery of a given active substance precisely to the affected area. In addition to controlled drug release at a precise dose, the beneficial aspects of DDS include improved solubility of poorly water-soluble drugs, as well as high drug loading or delivery of multiple therapeutic agents in a single dose [1,2]. Structures widely used as drug carriers include micelles [3,4,5], liposomes [6,7,8], dendrimers and nanoparticles [9,10,11], which are characterized by high stability and also improve drug bioavailability and reduce dosing frequency [12].

Scaffolds used for tissue formation are required to meet several fundamental criteria. These include high porosity as well as sufficiently large pore size. In addition, it is crucial to have specific surface properties that will allow cellular tissue to adhere, differentiate and proliferate, as well as maintain the tissue structure and its ability to cooperate with the organism [13]. Furthermore, any bone substitute is expected to mimic the characteristics of natural bone, which will support osteoregenerative processes [14]. One frequently used inorganic carrier is calcium phosphate ceramic (CaP), especially hydroxyapatite (Ca_10_(OH)_2_(PO_4_)_6_, HA). Synthetic hydroxyapatite crystallizes in a hexagonal arrangement. Its density is 3.156 g/cm^3^, decreasing to 3.120 g/cm^3^ after heat treatment due to water loss [15]. It is a natural component of bone as well as teeth and has excellent mechanical properties [16]. Due to its excellent biocompatibility, bioactivity and biodegradability, it has found a wide range of applications in tissue engineering and medicine [17,18]. CaP-based materials promote osteoblast adhesion and proliferation [19,20]. Considering its large surface area and adsorption capacity, it can be a carrier for various drugs, including antibiotics [21,22]. Most commonly, HA is used in a porous form, which allows for a biological connection to the tissue, and is widely used as an implant material. The network of pores in the HA structure influences the proliferation of tissue cells. Moreover, blood vessels (e.g., capillaries) can grow into them during the formation of new bone or during the healing process. Such a procedure reinforces the implant in the target location as well as minimizes the risk of displacement [23].

Among the methods for obtaining hydroxyapatite, two groups can be distinguished, i.e., dry methods and wet methods. Dry methods consist of roasting calcium phosphate mixtures with a Ca/P molar ratio of 1.67 in a water vapor atmosphere at temperatures above 1000 °C. However, the most commonly used methods are wet methods, which involve precipitation from suspensions or aqueous solutions of calcium phosphates with a molar ratio of Ca/P equal to 1.67. In some cases, the hydroxyapatite obtained is subjected to roasting at temperatures above 1000 °C [24,25,26]. One of the advantages of synthetic HA is that its microstructure and the pore volume itself during synthesis can be controlled. This gives the possibility to adjust the surface of the hydroxyapatite depending on the application [27,28]. The hydroxyapatite obtained is often processed through a drying method to obtain a solid ceramic powder. Among the ceramic drying methods are atmospheric drying, drying under vacuum and the freeze-drying process. The latter technique is an advanced drying method, where ultra-fine crystalline particles with good dispersibility are obtained [29]. Freezing causes the solids in solution to be excluded by advancing ice into the intergranular spaces between the ice crystals. Subsequent sublimation then leads to the formation of porous structures that can carry various active substances [30].

One possibility for obtaining an HA-based drug carrier is to load it with various active substances, including antibiotics, for example, clindamycin. This drug is a common antimicrobial agent used to treat diseases where bone loss has occurred [31,32]. Clindamycin belongs to the lincosamide group. Moreover, it is active against both anaerobic as well as aerobic Gram-negative and Gram-positive bacteria [33,34,35]. Clindamycin is an antibiotic that plays an important role in implantology, particularly in the context of preventing and treating infections associated with dental implants or other surgical procedures [36,37,38,39].

The following paper investigates the effect of the drying technique of synthesized HA on the porosity of ceramic powders as potential carriers of active substances. The obtained ceramic powders were subjected to physicochemical analysis including density testing, X-ray diffraction analysis (XRD), Fourier-transform infrared spectroscopy (FTIR) and scanning electron microscopy (SEM). Furthermore, the sinters formed were modified with clindamycin. Using the high-performance liquid chromatography (HPLC) technique, the rate of drug release was determined. The analyses carried out allowed assessment of the potential of the developed materials as drug carriers for targeted therapy. To the best of our knowledge of the literature, there are no studies of identically obtained material combinations.

## 2. Materials and Methods

### 2.1. Reagents

The reagents used for the synthesis of hydroxyapatite, including phosphoric acid (HNO_3_), calcium hydroxide (Ca(OH)_2_) and ammonia water (NH_4_OH, 25%), were purchased from Sigma-Aldrich (Darmstadt, Germany). The antibiotic used for the modification was clindamycin hydrochloride, purchased from Sigma-Aldrich (Darmstadt, Germany). The mobile phase used for HPLC was a combination of acetonitrile (CH_3_CN) from Honeyweel (Seelze, Germany) and potassium dihydrogen phosphate (KH_2_PO_4_) from DOR-CHEM (Krakow, Poland). Phosphate-Buffered Saline Tablets were purchased from OXOID (Basingstoke, UK). A Hydrolab model HLP 5sp unit was used to obtain demineralized water for all solutions.

### 2.2. Preparation of Hydroxyapatite

Hydroxyapatite (HA) was obtained by a wet precipitation method by reacting H_3_PO_4_ and Ca(OH)_2_. To obtain HA, 500 mL of 0.3 mol/L aqueous solution of H_3_PO_4_ was dropped into a stirring aqueous solution of 500 mL of 0.5 mol/L Ca(OH)**_2_** at a rate of 1 drop/sec. The pH of the mixture was maintained at about 11 with 25% ammonia water. After the reaction was completed, the reaction mixture was aged for 24 h. After this time, the mixture was washed with distilled water to the natural pH and filtered through a fluted filter. This method was also described in the previous article [40]. Two syntheses were carried out in an analogous manner. The first batch of hydroxyapatite powder was subjected to drying in a POL-EKO model dryer SLW 400 (Wodzisław Śląski, Poland) at 104 °C for 4 h, and it was designated d-HA. The second part was dried in a Martin Christ Alpha 1–2 LDplus freeze dryer (Osterode am Harz, Germany) at −50 °C for 48 h and was identified as f-HA.

From the resulting hydroxyapatite powders dried by both methods, disks were obtained by pressing 0.5 g of ceramic powder under 4 tons of pressure. Sintering was then performed by annealing the material for 2 h at 1000 °C.

### 2.3. Determination of Density

Density was determined using a pycnometer with a capacity of 25 cm^3^. After rinsing with acetone and drying, the pycnometer was weighed, and the measurement was re-peated after filling it with distilled water at the temperature of 22 °C. After drying, the pycnometer was filled with 5 g of hydroxyapatite powder, which had previously been sifted through a 0.2 mm mesh sieve, and weighed again. The next step was to pour water over the pycnometer with the powder to about 15 mm above. In order to remove the air from the powder layer, it was put in the dryer for about 20 min. After being taken out and cooled, it was refilled with water, and a final weighing was performed. Measurements were made for dried and freeze-dried hydroxyapatite. The true density was calculated from the formula:

(1) $$d_{rz}=\frac{m_{2}-m_{1}}{\frac{m_{3}-m_{1}}{d_{0}}-\frac{m_{4}-m_{2}}{d_{0}}}=\frac{d_{0}\cdot \left(m_{2}-m_{1}\right)}{\left(m_{2}-m_{1}\right)-\left(m_{4}-m_{3}\right)}$$

where:

*m*_1_—mass of empty pycnometer (g),
*m*_2_—mass of pycnometer with powder (g),
*m*_3_—mass of pycnometer filled with water (g),
*m*_4_—mass of pycnometer with powder filled with water (g),
*d*_0_—density of water at measurement temperature (g/cm^3^),
*d_rz_*—actual density of hydroxyapatite (g/cm^3^).

### 2.4. X-ray Diffraction Analysis

The resulting hydroxyapatite was subjected to structural characterization by X-ray diffraction (XRD). For this purpose, a Malvern Panalytical Aeris X-ray diffractometer was used with a PIXcel1D-Medipix3 detector (Malvern, UK) using Cu Kα radiation. Measurements were carried out at a step size of 0.0027166° 2θ over a range of 2θ from 10 to 100° with a time per step of 340.425 s. Measurements were analyzed as described previously [41].

### 2.5. Fourier-Transform Infrared Spectroscopy Analysis

Fourier-transform infrared spectroscopy (FT-IR) was used to identify characteristic functional groups in the synthesized hydroxyapatite. Using a Thermo Scientific Nicolet iS5 FT-IR spectrometer (Thermo Scientific, Loughborough, UK) equipped with an iD7 ATR accessory operating under room conditions in the range of 4000 cm^−1^–400 cm^−1^ (32 scans at 4.0 cm^−1^ resolution), spectra were analyzed for the resulting ceramic powders as previously described [42].

### 2.6. Determination of Average Grain Diameter

The sedimentation rate of ceramic powders in water was determined using a MultiScan MS20 DataPhysics instrument (Stuttgart, Germany). In this technique, the transmission of monochromatic NIR radiation (wavelength of 100 nm) is measured by using a transmission detector that determines the radiation transmitted by the sample. As a result of the measurement, the sedimentation rate of particles in water as well as their average diameter can be identified. The sedimentation rate and the resulting transmission provide information about the homogeneity of the substance, defining whether the grain diameters are approximated [43]. For the measurement, 0.5 g of powder was placed in a vessel filled with 30 mL of distilled water. The test was performed for 5 min, with 20 readings (interval of 15 s) conducted at 23 °C. The examination was carried out for oven-dried hydroxyapatite as well as analogously for freeze-dried powder.

### 2.7. Synthesis of Clindamycin-Modified Hydroxyapatite

In order to provide the obtained materials with the character of an active substance carrier, the prepared 0.5 g disks were subjected to physical sorption in a solution of the clindamycin hydrochloride. The disks were placed in the 10 mL of drug solution with a concentration of 2 mg/mL for 24 h at 4 °C in a laboratory refrigerator. The process was carried out based on the phenomenon of physical sorption and carried out for oven-dried hydroxyapatite and analogously for freeze-dried one. Thus, a ceramic drug carrier was prepared. The carriers were designated as C/d-HA for dryer-dried hydroxyapatite and C/f-HA for freeze-dried powder.

### 2.8. Kinetic Release Studies of Antibiotic

The rate of drug release from the ceramic carriers was determined. For this purpose, the drug-modified sinters were placed in sterile, screw-top containers containing 30 mL of PBS solution. The containers were then placed in a POL-EKO thermostatic cabinet, model ST 5 B SMART (Wodzisław Śląski, Poland). Measurement of drug concentration in PBS was performed after 3 h and then after 1, 3, 5 and 7 days. The amount of clindamycin hydrochloride released was determined by high-performance liquid chromatography (HPLC) using equipment from Shimadzu (Kyoto, Japan). Mobile phase was composed of acetonitrile (45%) and potassium dihydrogen phosphate (55%, pH 7.5). The measurement was carried out for 11 min at 30 °C and 90 bar analogously, as described earlier [44]. Two repeats were performed for each composition, and the results were averaged.

### 2.9. Morphology Analysis

Surface morphology analysis studies of the obtained samples were carried out on a Jeol 5510LV scanning electron microscope (SEM, Jeol, Freising, Germany). Prior to the measurement, nano-gold was sputtered onto the surface of the samples using a vacuum sputtering machine (Cressington, model 108 auto, Hill Watford, UK). The study was carried out for both pure hydroxyapatite powders and sinter to compare changes in surface morphology depending on drying methods [45].

## 3. Results

### 3.1. Determination of Density

The calculated density for d-HA and f-HA powders is demonstrated in Table 1. The difference in density measurements was small, around 9%. HA dried in a dryer had a higher value than freeze-dried HA.

### 3.2. X-ray Diffraction Analysis

**Figure 1:**
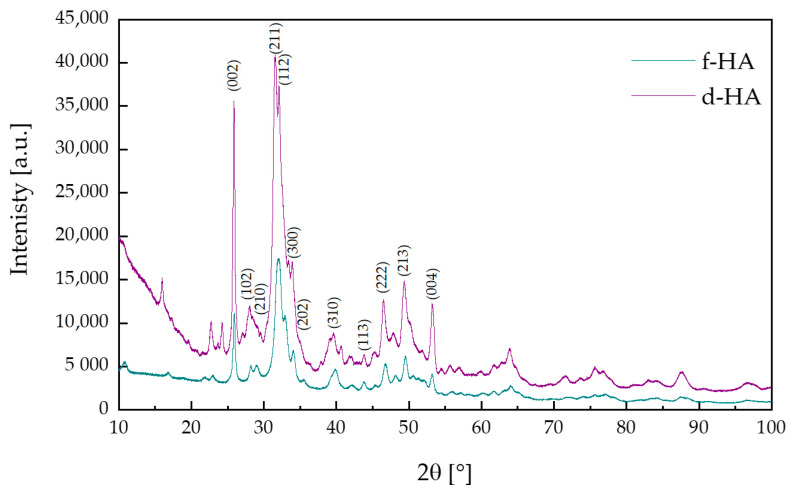
X-ray diffraction (XRD) patterns of f-HA and d-HA powders.

The diffractogram pattern of the synthesized hydroxyapatite for samples f-HA and d-HA is illustrated in Figure 1. According to International Centre for Diffraction Data sheet no. 01-080-7085, the diffraction reflections obtained are attributed to hydroxyapatite with a hexagonal structure (P63/m space group), as confirmed by the characteristic peak at 31.94 two theta (211) [40,41].

The degree of crystallinity (*X_C_*) of the ceramic powder was determined according to the formula:

(2) $$X_{C}=\frac{P_{C}}{P_{C}+P_{A}},$$

where the crystalline fraction was determined by integrating the areas under crystalline (*P_C_*) and amorphous (*P_A_*) diffraction. This parameter was calculated using OriginPro 2023 (10.0) software.

For the synthesized ceramic powder designated f-HA, the degree of crystallinity was 60.7%, while for the ceramic powder marked as d-HA, it equaled 51.9%. The background of the XRD spectrum of the dried ceramic powder was larger than for the ceramic powder dried by freeze-drying. Furthermore, the selected drying technique for the resulting ceramic powder also influenced the degree of crystallinity of the test sample. Based on the results obtained, in both samples hydroxyapatite was the only phase identified. In addition, it was confirmed using XRD that crystalline and pure ceramic powders are obtained by the wet synthesis method for calcium phosphate ceramics.

### 3.3. Fourier-Transform Infrared Spectroscopy Analysis

**Figure 2:**
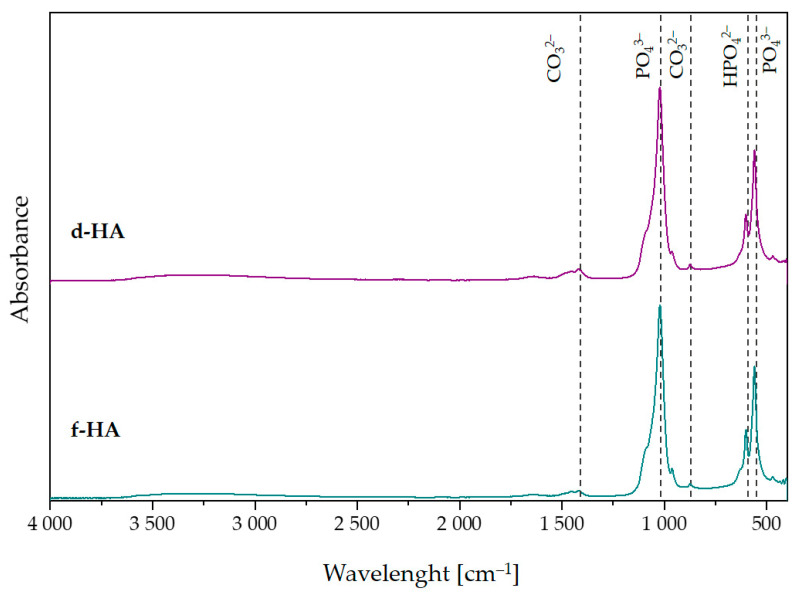
FT-IR spectra of powders: d-HA and f-HA.

Figure 2 compares the FT-IR spectra obtained for hydroxyapatite dried in a dryer and in a freeze-dryer. Both FT-IR spectra showed characteristic peaks attributable to hydroxyapatite. At both wavelength lines of 1090 cm^−1^ and 1024 cm^−1^, there were peaks attributable to the ν_3_ asymmetric stretching mode of P–O [40]. The ν_4_ triply degenerate bending mode of PO_4_^3−^ (O–P–O) can be attributed to the peaks occurring at 558 cm^−1^ and 600 cm^−1^ [46]. In addition, both in the d-HA and f-HA samples, there were characteristic peaks originating from the ν_3_ stretching mode of CO_3_^2−^ at 1453 cm^−1^ [40,47]. Furthermore, at 1410 cm^−1^, there was a peak attributed to ν_3_ carbonate ions and, at 874 cm^−^^1^, one attributed to the ν_2_ bending mode of carbonate ions [48]. The presence of these peaks may be due to atmospheric adsorption occurring after synthesis. In addition, the FT-IR spectrum also showed peaks at 631 cm^−^^1^, responsible for the δ in-plane vibration of OH^−^ [49,50].

### 3.4. Determination of Average Grain Diameter

Measuring the stability of suspensions and thus determining the sedimentation rate is a way to calculate the average grain diameter of ceramic powder. The rate of particle fall is affected by factors such as ambient temperature, medium viscosity and grain size [51]. The measurement was carried out for a 0.5 g powder sample suspended in distilled water at 23 °C (water density 997.66 kg/m^3^).

**Figure 3:**
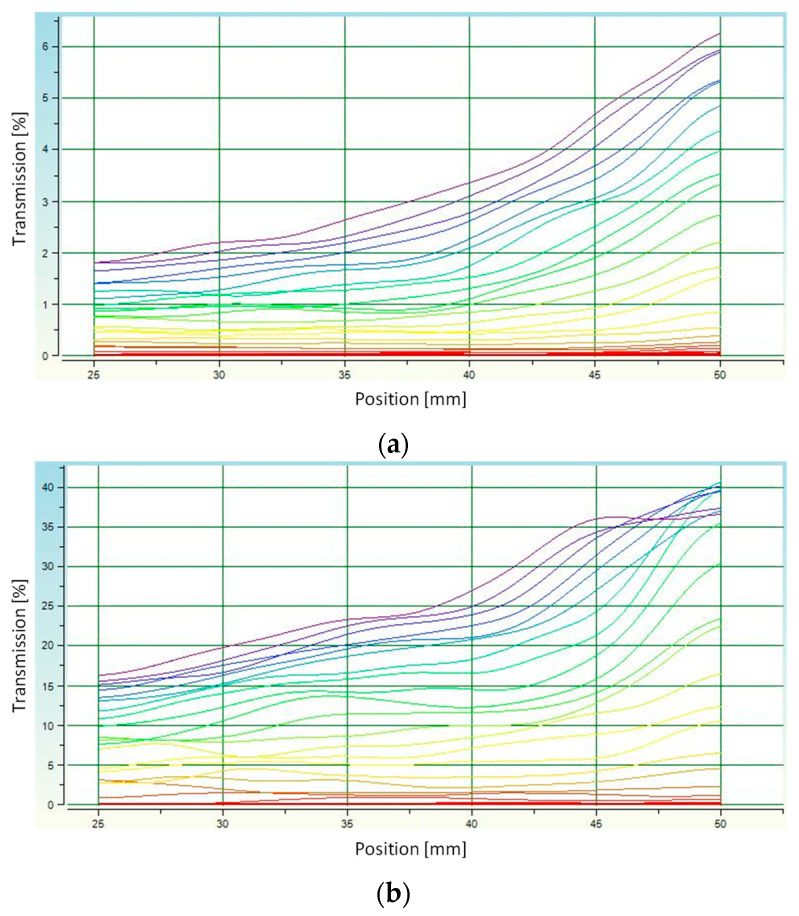
Analysis of the stability of hydroxyapatite suspensions in distilled water: (**a**) transmission of f-HA; (**b**) transmission of d-HA. Individual colors indict the next measurement (every 5 s).

The results of the sedimentation rate test are presented in Figure 3. The noticeable increasing value of transmission over time indicates the sedimentation process occurring. This increase was observed at the highest position values and indicates gradual clarification of the HA/H_2_O suspension in the upper part of the measuring vessel.

The shape of the curves in the transmission graphs indicates the monodispersity of the suspension. For f-HA, the curves on the plot are less wavy than for d-HA. This suggests that the grain sizes were more homogeneous for the freeze-dried material. It is likely that the low temperature as well as the high pressure set during the drying process prevented the formation of larger grain agglomerates. A slightly opposite situation can be observed for d-HA ceramics. In this case, the more wavy transmission lines suggest that sedimentation did not occur homogeneously throughout the volume of the measuring vessel. Thus, for f-HA, the analyzed suspension was more monodisperse than for d-HA. The determined sedimentation rate was −26.31 ± 0.76 mm/min for d-HA and −19.23 ± 0.02 mm/min for f-HA.

Determination of the actual density (Section 3.1.) enabled subsequent calculation of the average particle diameter of ceramic grains during the sedimentation rate test using the MultiScan MS 20 software. The average particle diameter of d-HA was ⌀ = 19.60 µm, while for f-HA it was ⌀ = 11.43 µm. This confirms the aforementioned conjecture that the faster sedimentation of grains in the case of d-HA was caused by their slightly wider diameter.

### 3.5. Kinetic Release Studies of Antibiotic

**Figure 4:**
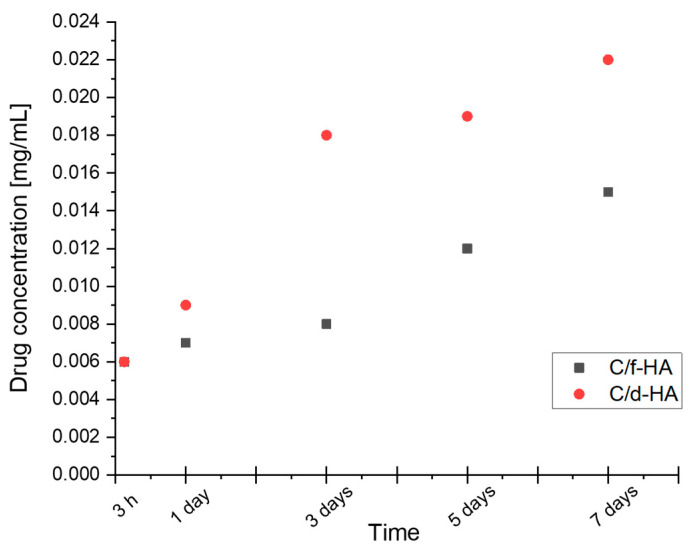
Clindamycin release from ceramic powders.

Determination of drug release from d-HA and f-HA powders was carried out for one week. A graph of clindamycin release rate in mg/mL in relation to time is presented in Figure 4. In order to distinguish drug-modified samples from those without the drug, the abbreviations C/d-HA and C/f-HA were introduced. The “C” designation symbolizes clindamycin loaded into the carrier.

The results of antibiotic release from C/d-HA and C/f-HA into 30 mL PBS are presented. The amount of clindamycin indicated on the *y*-axis refers to the mg/mL concentration. For this reason, the value should be multiplied appropriately for the amount of liquid in which the release was conducted. For both carriers, the same 0.18 mg amount of released drug was observed after 3 h. However, after just 1 day, differences were noticed in the rate of clindamycin entering the PBS solution, which persisted until the end of the study. Finally, after 7 days, 0.66 mg of drug was released from C/d-HA, while about one-third less (0.45 mg) was released from freeze-dried C/f-HA material.

**Figure 5:**
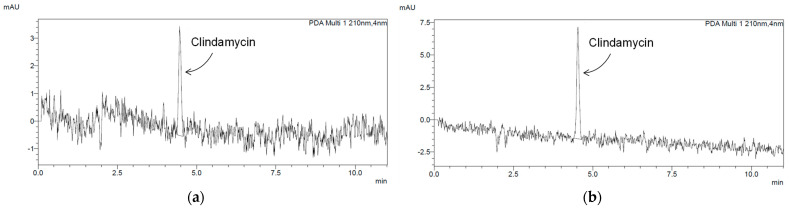

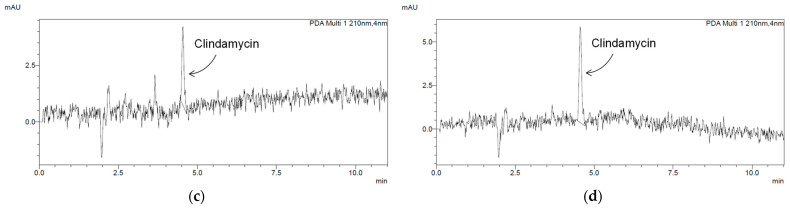
Chromatograms demonstrating clindamycin release: (**a**) sample C/d-HA after 1 day of incubation in PBS; (**b**) sample C/d-HA after 7 days of incubation in PBS; (**c**) sample C/f-HA after 1 day of incubation in PBS; (**d**) sample C/f-HA after 7 days of incubation in PBS.

These differences can be clearly observed in Figure 5, which demonstrates the chromatograms for day 1 and day 7. The peak for clindamycin was determined at retention times around 4500. It was observed that even after the first day of drug release testing, the milli-absorbance (mAU) value on the chromatogram was slightly higher for C/d-HA than for C/f-HA (Figure 5a,c). For the last, seventh day of release, the difference in intensity between the powders was even more noticeable (Figure 5b,d). Presumably, drug particles adsorbed on the outer surface of the ceramic disks were released into the PBS solution in the first moments of the study. The 7-day release was possible since drug molecules from inside the biomaterial entered the incubation medium with time. The molecules leached out as a result of PBS penetration into the pores of the disks. Noise on the background spectrum in the chromatograms suggests that other substances may have leached from the biomaterial in addition to the drug.

Therefore, it can be concluded that the rate of drug release from the ceramic carrier depends both on time as well as the type of powder (differences resulting from the drying technique). The concentration of the clindamycin in the PBS solution increased steadily over a period of 7 days.

### 3.6. Morphology Analysis

**Figure 6:**
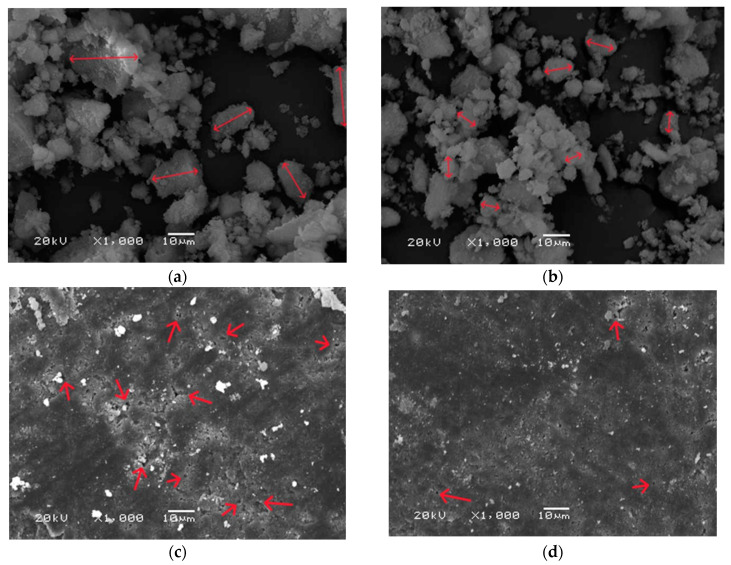
SEM surface morphology analysis of powders (**a**) d-HA and (**b**) f-HA and samples sintered at 1000 °C: (**c**) d-HA, (**d**) f-HA. The red arrows indicate the pores.

Figure 6 presents the structure of hydroxyapatite powders and sinters. The particles of powder from dryer (a) were larger, reaching diameters in a range of about 20 μm, and the grains had a more elongated shape than freeze-dried powder particles, which were more spherical and had smaller diameters in the range of 1–10 μm. In the images of both sinter samples, pores are present, and more were observed for the dried HA sinters. The number of pores is important for the sorption capacity of the material [52].

## 4. Discussion

As a conclusion, hydroxyapatite can be successfully obtained by the proposed wet synthesis method. The results indicate that the only phase identified was the hydroxyapatite phase. This is confirmed by the results of XRD analysis, which demonstrated that the ceramic powder obtained was phase pure, and the diffractograms corresponded to the data sheets (according to the ICDD). In addition, by observing the spectral background, it can be concluded that hydroxyapatite dried both in a dryer and using the freeze-drying technique is partially crystalline and amorphous. The degree of crystallinity was higher for the freeze-dried sample. Furthermore, FT-IR analysis confirmed the characteristic functional groups in the investigated ceramic powders dried using a dryer as well as a freeze-drying technique.

According to the HPLC results, freeze-dried hydroxyapatite released less clindamycin during incubation studies in PBS. It was also characterized by a smaller grain size than d-HA, as demonstrated by analysis of sedimentation rate and average grain diameter. Thus, it is possible that the d-HA agglomerates were able to bind a larger amount of clindamycin into their spaces or pores during physical sorption, which was then released over time. In addition, the apparent noise in the background spectrum on the chromatograms may indicate that other substances in addition to the drug may have leached from the biomaterial.

The method of drying hydroxyapatite does not significantly affect the change in density of the resulting powder; the differences in density measurements are small, but it affects the shape of the particles, as well as the porosity of the sinters made from it, as could be seen during SEM measurements. Moreover, when comparing the SEM grain size measurement with the average grain diameter size determined by sedimentation rate, the results are similar, which confirms that the measurements obtained using scanning electron microscopy were correct. The presence of pores is a positive phenomenon, because the more pores there are, the greater the amount of active substance that can be absorbed by the material. According to SEM analysis, more visible pores were present in the d-HA sinter compared to f-HA sinter. Comparing the clindamycin release results from the HPLC assay with the number of pores in the sinter confirms that the sinter with the higher pore count, d-HA, released more of the active substance.

The results presented in this manuscript confirm that the way hydroxyapatite is dried affects the particle shape as well as the porosity of the sinters prepared from it. Furthermore, the studies conducted indicate that the method of drying influences the sorption capacity of the hydroxyapatite sinters. Moreover, the amount of active substance released, in this case, clindamycin, from the hydroxyapatite sinters is related to the sorption capacity. Thus, it can be concluded that the synthesized porous ceramic powders have the potential to be successfully applied as active substance carriers in a drug delivery system. However, further mechanical and biological analysis is required.

## Figures and Tables

**Table 1:** Density of d-HA and f-HA powders (mean ± SD).

Symbol	Unit	d-HA	f-HA
d_rz_	g/cm^3^	2.69 ± 0.08	2.44 ± 0.12

## Data Availability

Data that support the findings of this study are contained within the article.
